# Anatomy of enzyme channels

**DOI:** 10.1186/s12859-014-0379-x

**Published:** 2014-11-18

**Authors:** Lukáš Pravda, Karel Berka, Radka Svobodová Vařeková, David Sehnal, Pavel Banáš, Roman A Laskowski, Jaroslav Koča, Michal Otyepka

**Affiliations:** National Centre for Biomolecular Research, Faculty of Science and CEITEC, Central European Institute of Technology, Masaryk University Brno, Kamenice 5, Brno-Bohunice, 625 00 Czech Republic; Department of Physical Chemistry, Regional Centre of Advanced Technologies and Materials, Faculty of Science, Palacký University Olomouc, tř. 17. listopadu 12, Olomouc, 771 46 Czech Republic; Faculty of Informatics, Masaryk University Brno, Botanická 68a, Brno, 602 00 Czech Republic; European Molecular Biology Laboratory, European Bioinformatics Institute (EMBL-EBI), Wellcome Trust Genome Campus, Hinxton, Cambridge, CB10 1SD UK

## Abstract

**Background:**

Enzyme active sites can be connected to the exterior environment by one or more channels passing through the protein. Despite our current knowledge of enzyme structure and function, surprisingly little is known about how often channels are present or about any structural features such channels may have in common.

**Results:**

Here, we analyze the long channels (i.e. >15 Å) leading to the active sites of 4,306 enzyme structures. We find that over 64% of enzymes contain two or more long channels, their typical length being 28 Å. We show that amino acid compositions of the channel significantly differ both to the composition of the active site, surface and interior of the protein.

**Conclusions:**

The majority of enzymes have buried active sites accessible via a network of access channels. This indicates that enzymes tend to have buried active sites, with channels controlling access to, and egress from, them, and that suggests channels may play a key role in helping determine enzyme substrate.

**Electronic supplementary material:**

The online version of this article (doi:10.1186/s12859-014-0379-x) contains supplementary material, which is available to authorized users.

## Background

Channels inside biomacromolecular structures (proteins, nucleic acids and their complexes) play many significant biological roles as they enable traffic between the interior spaces and the exterior. In enzymes they allow passage of substrates and products to/from the active site [1-12], in the ribosome they allow nascently synthetized proteins to pass from the proteosynthetic center to the outside [13], and in membrane proteins they provide high specificity of passage in either direction through the membrane [14,15]. Thus channels have attracted the attention of many researchers, who have rationalized their biological roles using a variety of experimental and theoretical methods. The ribosome, for example, prevents nascently synthetized polypeptides getting stuck in its polypeptide egress channel by lining the wall of the channel with a mosaic of alternating negative and positive electrostatic potentials [13,16]. Gramicidin provides polar holes for biomembranes, enabling free diffusion of monovalent ions and water through the membrane [17-19], while transmembrane ion channels maintain their high selectivity by a combination of structural and electrostatic features of the channel-lining amino acids [14,20].

Enzymes are proteins that catalyse reactions changing substrates to products. The enzymatic reactions occur in the enzymes’ active sites. Thanks to the many analyses of enzymatic reactions, we now have a better understanding of how active site chemistry works [21-24] and which amino acids are present in the sites [25]. However, relatively little is known about how substrates enter active sites and how the respective products leave them. While some active sites are positioned on the protein’s surface, in clefts or pockets, other enzymes have deeply buried active sites, which are connected to the outside by one or more channels. Here we focus on these channels, as the active site access paths play an important role in substrate and product trafficking between active site and outside. It has been shown that mutations in enzymes’ active site access channels alter the substrate preferences of haloalkane dehalogenase enzymes and may be utilized in rational design of enzymes [26,27]. The amino acids lining the access channels of cytochrome P450 (CYP) are important for the selectivity of these enzymes [28] while the flexibility of these channels, i.e. their opening and closing motions, contributes to the broad substrate specificity of CYP [10,29].

**Figure 1:**
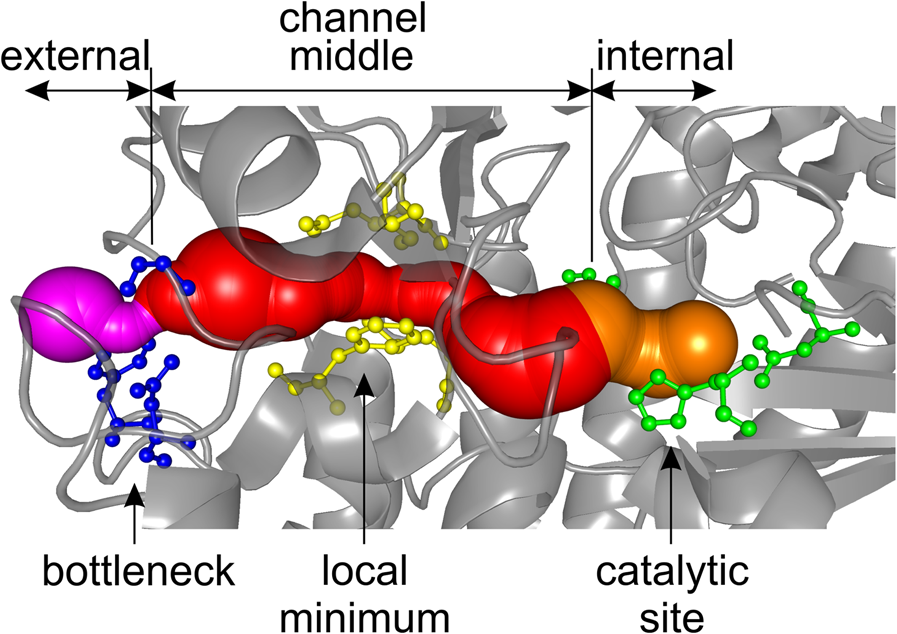
**An example of an enzyme channel identified by MOLE 2.0.** Internal, middle and external parts of the channel in pyridoxal-5'-phosphate-dependent acyl-CoA transferase (PDBID: 3KKI) are colored orange, red and magenta, respectively. Active site amino acids (present in the internal part of the channel) are shown in green, amino acids in the middle part making the wall of a local minimum (channel narrowing) are in yellow, and amino acids in the external part lining the bottleneck are in blue.

Despite a large effort, and recent progress in the field, an in-depth analysis of enzyme channels is lacking. Here, we use an advanced software tool, MOLE 2.0, developed for analysis of biomacromolecular channels [30], to survey 4,306 enzymes annotated in the Catalytic Site Atlas (CSA). We identify that more than 64% of enzyme structures contain channels at least 15 Å long from the active site. A typical enzyme channel is ~20 Å long and its walls are made preferentially of histidine, arginine, tryptophan and tyrosine residues and, to a lesser extent, by phenylalanine, asparagine, and aspartic acid (Figure 1). These residues can be considered as gate-keepers controlling the entry to and from the active site.

## Results

### Geometry features

We identified that at least 64.2% of enzymes contain channels at least 15 Å long (Table 1) and 86.8% contain channels at least 5 Å long. However, as the short channels may correspond to paths connecting cleft-like active sites with the exterior, we decided to use only channels longer than 15 Å for our analysis. Enzymes with channels (of ≥15 Å in length) leading to a buried active site contained on average two such channels (Table 2). Some had more than five such channels, the highest number being 68 in 6,7-dimethyl-8-ribityllumazine synthase from *Aquifex aeolicus* (PDBID: 1NQU; Additional file 1: Figure S1). Whereas one might expect larger proteins to contain larger numbers of channels, we found that the number of long channels does not correlate with protein size (Additional file 1: Figure S2). The median channel length is 27.7 Å (Table 2 and Additional file 1: Figure S3), 40% of channels are 15–30 Å long and 10% of enzymes contain channels longer than 50 Å. The longest channel (172 Å long) was found in Penicillium vitale catalase from *Penicillium janthinellum* (PDBID: 2IUF; Additional file 1: Figure S1). It should be noted that although the size of small enzymes (i.e. those containing fewer than 5,000 atoms) does not limit the number of long channels they contain, it does limit the maximum length these channels can have (Additional file 1: Figure S2).

**Table 1:** **Number of enzyme entries in each EC class, and numbers of channels of different lengths**

**EC**	**Enzyme class**	**Number of enzymes**	**Enzymes with channels of length**			
			**≥ 5 Å**	**≥10 Å**	**≥ 15 Å**	**≥ 20 Å**
EC1	Oxidoreductases	879	781	736	**684**	612
EC2	Transferases	1096	963	863	**747**	639
EC3	Hydrolases	1455	1228	1019	**749**	542
EC4	Lyases	465	401	361	**318**	262
EC5	Isomerases	252	226	204	**165**	140
EC6	Ligases	159	139	118	**98**	88
	Sum	4306	3738	3301	**2761**	2283

Numbers of enzymes in the dataset containing at least one channel of the given length. Bold values indicate 15 Å threshold for channel detection used thorough the study.

**Table 2:** **Geometrical channel features for all enzyme classes**

**EC**	**Na**	**P**	**M** ^**a**^	**L**
EC1	6518	77.8	3	29.8
EC2	4728	68.2	3	26.3
EC3	3454	51.8	2	27.5
EC4	5780	68.4	2	27.9
EC5	5107	65.5	3	26.4
EC6	5289	61.6	2	25.2
All	4823	64.2	2	27.7

^a^Enzymes not containing channels were excluded.

Average number of atoms (Na), percentage of enzymes containing at least one channel from the active site longer than 15 Å (P in %), median number of channels (M), and median length of channels (L in Å) for each enzyme class.

**Figure 2:**
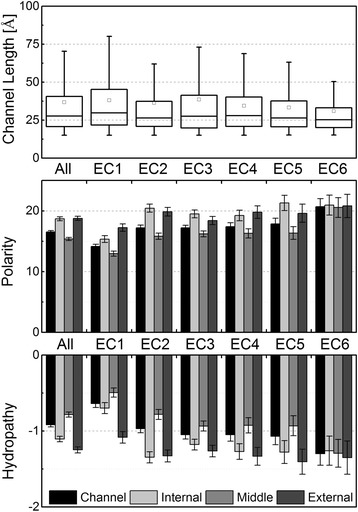
**Properties of channels in different enzymatic classes.** The topmost panel shows the variability of average channel length in individual enzymatic classes in comparison with the overall average channel length. The middle panel shows the average channel polarity for each enzyme class as well as the polarity of individual parts of the channels. The colors of the bars correspond to different parts of the channels, as shown in the key at the bottom. The bottom panel shows the variability in average hydropathy. Error bars show the standard error of the mean.

Channel occurrence and length varies among the enzymatic classes (Table 2). The highest percentage (77.8%) of proteins with channels longer than 15 Å was identified in oxidoreductases (EC1), while the lowest percentage (51.8%) applied to hydrolases (EC3). The number of channels is slightly elevated in oxidoreductases (EC1), transferases (EC2) and isomerases (EC5). Oxidoreductases (EC1) have median channel length longer by about 2 Å than other enzymes, whereas transferases (EC2) and ligases (EC6) have average channel length shorter, also by about 2 Å (Figure 2). As a result, oxidoreductases stand out of the crowd, as they have both higher channel occurrence and longer channels (Additional file 1: Figure S2).

### Physico-chemical features

For each channel, we calculated basic physico-chemical features such as hydropathy [31] and polarity [32] using the method introduced in our previous paper [30]. In brief, the method sums up the length-weighted physico-chemical properties of the amino acids lining the channel. As the algorithm is rather approximate, we would like to note that the estimated physico-chemical properties should be interpreted with care. The average channel hydropathy is −0.92 (Table 3) and this value is close to the hydropathy of tyrosine and tryptophan (it is worth noting that the hydropathy of amino acids varies from −4.5 of Arg to 4.5 of Ile). The distribution plots of hydropathy (Additional file 1: Figure S3) also indicate that the values are shifted in most channels to negative values indicating that hydrophilic channels are more preferred over hydrophobic ones.

**Table 3:** **Physicochemical features of channels**

**EC**	**Hydropathy**	**Polarity**	**N(+)**	**N(−)**	**Δ**
EC1	−0.64 ± 0.05	14.2 ± 0.4	3	2	1
EC2	−0.97 ± 0.06	17.2 ± 0.4	3	2	1
EC3	−1.05 ± 0.05	17.2 ± 0.4	3	2	0
EC4	−1.05 ± 0.08	17.4 ± 0.6	3	2	1
EC5	−1.07 ± 0.11	17.9 ± 0.9	3	2	1
EC6	−1.30 ± 0.15	20.7 ± 1.3	4	2	1
**All**	**−0.92 ± 0.03**	**16.5 ± 0.2**	**3**	**2**	**0**
Internal	−1.11 ± 0.04	18.7 ± 0.3	1	1	0
Middle	−0.78 ± 0.03	15.4 ± 0.2	1	0	0
External	−1.25 ± 0.04	18.8 ± 0.3	1	1	0

The average hydropathy and polarity are shown for each enzyme class as well as for the internal, middle and external parts of channels. Also shown are the median number of charged amino acid side chains lining the channels (N(+): positive Arg, Lys and His, N(−): negative Asp and Glu, and Δ: median of overall charge). Bold values indicate features of all channels.

The average channel polarity is 16.5, which falls between the values of highly polar amino acids (Asp, Glu, Lys, Arg and His having polarities of 49.5 – 52.0) and those of other amino acids (with polarities of 0.0 – 3.5). It indicates that the channels are rather polar as well as hydrophilic. Taking all this information into account we may conclude that the average channel has slightly negative hydropathy and higher polarity. However, highly hydrophobic and nonpolar, as well as highly hydrophilic and polar, channels were also detected. We also analyzed the presence of charged amino acid side chains (Asp, Glu, His, Lys and Arg) in channels walls (Additional file 1: Table S2). On average the channel walls are lined by two negative and two positive side chains resulting in sum neutral channel walls (Table 3).

We also identified channels with significant extreme physico-chemical properties (Additional file 1: Table S3 and Additional file 1: Figure S1). Here we present two examples. A highly hydrophilic channel (hydropathy index −3.8) of length 18.7 Å occurs in 3-deoxy-D-arabino-heptulosonate-7-phosphate synthase (PDB ID: 1N8F) from *E. coli*. The high hydrophilicity of the channel is in accord with its function [33] since this enzyme catalyses a condensation reaction between two highly polar substrates: phosphoenolpyruvate and erythrose-4-phosphate. It is worth noting that transferases (EC2) show the largest variability in hydrophobicity as illustrated by the fact that six times transferases are in the top 10 having highly hydrophilic channels, and four occur in the top 10 with highly hydrophobic channels.

At the other extreme is peroxidase (PDBID: 1LYK) from the fungus *Coprinus cinereus* [34] which has a highly hydrophobic channel (hydropathy index of 3.59) of length 23.8 Å. This channel enables transport of simple phenols and smaller aromatic dye molecules for their oxidation in lignin decomposition [35] in a process which has been exploited in biotechnology as a dye-transfer inhibitor in a laundry detergent [36].

These examples show that enzymatic classes differ in their average physico-chemical properties: (i) oxidoreductases (EC1) show the most hydrophobic as well as the least polar channels among the enzyme classes, while (ii) ligases (EC6), and to some extent also isomerases (EC5), lyases (EC4) and hydrolases (EC3), show the most hydrophilic as well as the most polar channels (Figure 2, Table 3 and Additional file 1: Table S3).

We also identified that some physicochemical features differ across the three channel layers: internal, middle and external (Table 3). The polarity of middle part of the channel is always lower than polarity of both internal and external parts, respectively. The lower polarity of the middle part of channel is also reflected by its significantly more hydrophobic behaviour. The charged residues occur mainly in external parts of enzyme channels, while the internal and middle part contains more aromatic residues.

### Channel-lining residues

**Figure 3:**
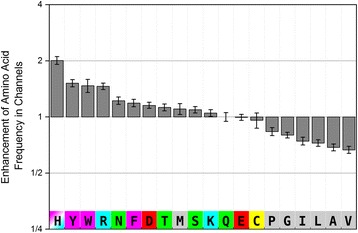
**Channel propensity of amino acids.** Enhancement of frequencies of individual amino acids in channels indicates which amino acids are more likely to occur in the channel walls than anywhere else in the protein structure. Hydrophobic aliphatic residues are shown in gray, aromatic amino acids in magenta, polar residues in green, negatively charged in red, positively charged in blue and cysteine in yellow.

We calculated the frequencies of channel-lining amino acids and compared them with frequencies of amino acids in the same enzyme structures. On the basis of this data, the channel propensities of individual amino acids can be defined as a ratio of the frequency of amino acid in the channel walls to the frequency of amino acid anywhere within the protein structure. The resulting channel propensities of the individual amino acids differ significantly. The rather bulky and aromatic amino acids (His, Tyr, Trp, Arg), occur over 1.25 times more frequently in the channel walls than in the whole enzyme. Additionally, other amino acids (Asn, Phe, Asp, Thr, Met, Ser) also show a slightly higher frequency in the channel walls than in the rest of the protein. Conversely, nonpolar aliphatic amino acids (Pro, Gly, Ile, Leu, Ala, and Val) are significantly less localized in channel walls (Figure 3). We also looked at the amino acid composition at each channel’s local minimum. Whereas this reflected the composition of the whole channel, the channel bottlenecks contain significantly more cysteine (Cys), histidine (His) and tyrosine (Tyr) residues than usual and much fewer small aliphatic amino acids (Pro, Gly and Ala) (Additional file 1: Figure S4). As histidine (His) and cysteine (Cys) have unique binding properties, it is possible to hypothesize that these binding properties might provide a gate-keeping activity at the channel bottlenecks, whereas small aliphatic residues cannot undergo large changes and as such cannot serve as gate-keepers.

**Figure 4:**
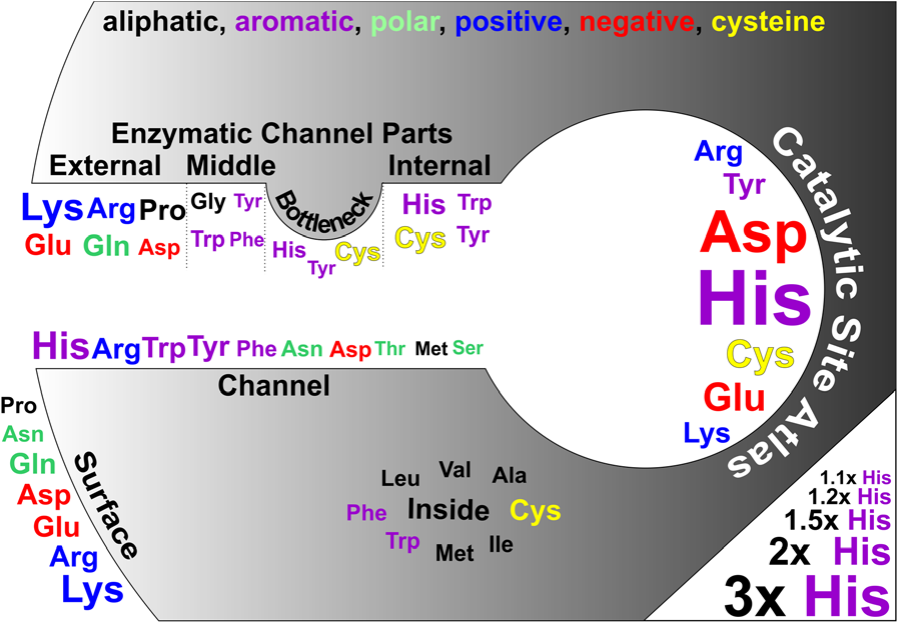
**Enhancement of amino acid frequency in different parts of the enzyme structure.** Amino acids that are found more often than average in different regions of an enzyme structure. Their labels are scaled to reflect their propensity for each compartment; the key in the bottom right-hand corner indicates how the label size relates to propensity. Hydrophobic aliphatic residues are shown in gray, aromatic amino acids in magenta, polar residues in green, negatively charged in red, positively charged in blue and cysteine in yellow.

The frequencies of amino acids in active sites, on the protein surface and inside the protein, or in general channels, are markedly different from both the average protein amino acid composition and the composition of the channels (Figure 4). The active sites contain significantly more amino acids that can be part of a catalytic cycle (His, Asp, Cys, Glu, Arg, Tyr, Lys) enabling proton and electron shuffling and covalent bond reorganization. On the other hand, the frequency of less reactive amino acids (Trp, Thr, Gln, Phe) or amino acids with nonreactive side-chain (Met, Ala, Pro, Ile, Val, Leu) is lower in the active sites. These results are in perfect agreement with data published by Holliday and coworkers [37]. Protein cores are rich in hydrophobic aliphatic (Ile, Leu, Val, Ala, Met) and aromatic amino acids (Phe, Trp) as well as in cysteines (Cys). These amino acids have a structural function to maintain the formation and stability of protein hydrophobic cores [38] and the formation of disulphide bonds [39]. Conversely, the surface regions contain mainly charged (Lys, Arg, Glu, Asp) and polar residues (Asn, Gln), which facilitate contact with the polar water environment. Also, the surface has a higher than average frequency of prolines (Pro) as these helix-breaker amino acids are common in turns in the protein structures rigidifying the protein fold (Additional file 1: Figure S4).

Channel-lining amino acids are not uniformly distributed along the length of the channel. Internal parts of the channel tend to contain more aromatic residues (His, Trp, Tyr) together with cysteine (Cys), while glutamine and glutamate (Gln and Glu) and aliphatic amino acids (Pro, Ala, Leu) are underrepresented. These trends are similar to the catalytic site propensities, as discussed in the previous paragraph. The frequencies of amino acids in the middle regions of the channels correspond to the frequencies in the entire channel with the exception of glycine (Gly) and aromatic amino acids (Trp, Tyr, Phe), which are present more frequently. We may hypothesize that the higher frequency of glycine (Gly) in the middle channel parts is because it facilitates flexibility, which may be important for substrate/product channeling between the active site and protein surface [29], whereas aromatic amino acids can serve as gate-keepers. External parts of the channel bear more charged residues than any other part (Arg, Lys, Glu, Asp) together with proline (Pro) and glutamine (Gln), whereas other polar residues (Ser, Thr) are surprisingly less common in the external parts even though that both Ser and Thr are evenly distributed within the structure of proteins (Additional file 1: Figure S4). External parts of channels are less occupied by aliphatic (Ile, Ala, Val) and aromatic amino acids (Phe, Tyr) as well as typical catalytic amino acids – histidine (His), aspartate (Asp) and cysteine (Cys). A higher frequency of charged or rather bulky and aromatic amino acids in channels may have functional implications because such amino acids may work as gate-keepers, regulating traffic between active site and surface via conformational changes. It is worth noting that some mutations of such residues have been shown to alter the catalytic efficiency and substrate preferences in haloalkane dehalogenases [26].

All the results above concern channels leading to buried enzyme active sites. For comparison, we also analyzed all channels of length greater than 15 Å connecting a cavity in the protein interior to the outside exterior (Additional file 1: Figure S4). We compared the amino acid compositions of the two types of channels. The active site access channels have higher frequencies of aromatic (Tyr, Trp and Phe), polar amino acids (Asn, Thr and Ser), catalytically active amino acids (Cys and His), and glycine (Gly). On the other hand, they contain fewer aliphatic amino acids (Pro, Leu, Ile and Val), charged and some polar amino acids (Lys, Glu, Arg and Gln). In sum, the active site access channels contain more functional amino acids than generic channels. This finding agrees with the idea that some amino acids bear functional roles in channels, e.g., as gate keepers or to maintain their flexibility.

The analysis of amino acids leading to active sites, divided according to the six enzymatic groups, shows that amino acid channel propensities correspond to overall channel propensities. However, some differences were identified (Additional file 1: Figure S4). As can be expected from their higher hydropathy and lower polarity, channels in oxidoreductases (EC1) have significantly lower frequencies of charged lining amino acids (Arg, Asp, Lys, Glu), but higher frequencies of aliphatic lining amino acids (Met, Pro, Ile, Leu, Ala, Val). Channels in transferases (EC2) contain fewer aromatic (Trp) and more charged (Arg, Asp, Lys) amino acids. Channels in hydrolases (EC3) contain fewer arginine (Arg), threonine (Thr) and aliphatic amino acids (Pro, Ile, Ala, Val), whereas they contain more aromatic (Trp, Tyr) and smaller charged (Lys, Asp, Glu) amino acids. Channels in lyases (EC4) show only lower amounts of aromatic (Trp) and sulphur containing (Met, Cys) amino acids. Channels in isomerases (EC5) contain fewer glycines (Gly). Channels in ligases (EC6) are the most hydrophilic channels, so it is not surprising that their channels contain fewer cysteine (Cys), aromatic (Trp, Tyr) and aliphatic (Pro, Leu) amino acids and more charged (Arg, Lys, Glu) amino acids and glycine (Gly). It should be noted that the differences between the individual enzyme classes should be interpreted with care because of larger statistical error bars, especially in the case of less populated EC5 and EC6 classes.

## Discussion

Long channels (>15 Å) are a common feature of enzymes, with over 64% containing at least two such channels. This shows that the majority of enzymes have buried active sites accessible via a network of access channels. Hence there is an apparent tendency for enzymes to bury their active site, i.e., to limit and control direct connection of active sites with the surrounding environment. This may be the result of two evolutionary pressures; i) steric, because active sites have to be structurally well arranged – a buried active site enables full spatial arrangement better than a pocket-like active site can give that half of the space of the latter is open to surrounding environment and ii) functional, as active site access paths may enable pre-selection of substrates, and may be involved in features co-determining enzyme substrate preferences. In another words, the active site access channels may limit access to the enzyme active sites and function as keyholes, enabling passage only of some classes of substrates.

The amino acid frequencies in the whole protein structures and channel walls differ significantly. Aliphatic amino acids are more involved in the formation of enzyme hydrophobic cores, which are important to maintain a protein fold. In turn, they are less frequently involved in channel wall lining or within the active sites. The aromatic, charged and polar amino acids occur more frequently in the channels walls. In addition, we identified a higher frequency of glycine in the middle parts of channels, which may function here to support channel flexibility enabling the channelling of bulkier substrates to active sites. This finding can be explained by the fact that the polar and charged amino acids line the channels to enable passage of polar substrates/products and water. The enhanced frequency of rather bulky and aromatic amino acids in channel external parts may have functional implications, because such amino acids may work as gate-keepers, regulating traffic between active site and outside.

The functional implications deduced from these global analyses are also supported by the fact that individual enzyme classes differ in their channel features. Typically oxidoreductases have the most hydrophobic, the least polar and longest channels among the enzyme classes, while ligases have the most hydrophilic, the most polar and the shortest channels. This indicates that evolution of enzymatic substrate preferences might also include evolution of active site access channels.

## Conclusions

To conclude, we analyzed channels in 4,306 enzyme structures annotated in the Catalytic Site Atlas. We identified that at least 64% of enzyme structures contain on average two channels longer than 15 Å leading to the catalytic site. Consequently, we may anticipate that the same number of enzymes have buried active sites. The longest, and also the most hydrophobic, channels are found in oxidoreductases, while the smallest number of channels can be found in hydrolases and the shortest and also the most hydrophilic channels in ligases. The composition of channel walls differs from the average composition of enzyme structures as well as from the composition of the protein surface. Hydrophobic aliphatic amino acids, which are the most common amino acids present in protein cores, occur in channel walls less frequently, whereas aromatic, charged and polar amino acids occur more frequently in channel walls. All these findings indicate that the active site access channels bear significant biological function as they are involved in co-determining enzyme substrate preferences.

## Methods

### Dataset

We analyzed 4,306 enzymes which were annotated in the Catalytic Site Atlas (CSA) database release of 4th March 2013 [25,40]. The dataset contained structures determined by X-ray diffraction at a resolution better than 2.5 Å, and had no two structures with a sequence identity higher than 90% (more quality checks can be found in Additional file 1: Table S1). It should be noted that when we used a dataset containing structures with a sequence identity less than 50%, the results did not significantly differ from the results obtained with the dataset containing structures with a sequence identity less than 90%. The enzymes in the dataset were grouped according to their Enzymatic Commission (EC) class (Table 1).

### Channel Identification

An active site is a cavity, which walls contain amino acids residues annotated in the CSA. A channel is a pathway inside an active site cavity connecting the deepest apex of the cavity with an exterior. The MOLE 2.0 program [30] was used for channel identification and characterization. Briefly, the MOLE 2.0 algorithm calculates the Delaunay triangulation/Voronoi diagram of the atomic centers, splitting it into several smaller parts and identifying suitable start and end points in the interior and surface, respectively. Dijkstra's algorithm is used to identify tunnels as the shortest paths between the start and end points (see Additional file 1 for further details). This algorithm is used also in the MOLEonline 2.0 web application [41]. The setup of MOLE 2.0 for these calculations was as follows: Probe Radius and Origin Radius 5 Å, Interior Threshold 1.1 Å and default values for Bottleneck Length, Bottleneck Radius, Cutoff Ratio and Surface Cover Radius. The CSA active sites were used as starting points. We used biological assemblies for the enzymes structures, which were obtained from the PDB database as *.pdb1 files. [42] Ten structures of EC 3.6.4 group were removed from the dataset. Hydrogen atoms and ligands not covalently bound to the structure were deleted prior to calculation. In cases where the system contained more than one active site, the site having the most channels was used. In order to study only relevant channels, we analysed only those channels longer than 15 Å. Table 1 shows the numbers of channels of different lengths for each of the six different enzyme classes, which are listed together with their properties in Additional file 2.

In the text we use the following terminology (Figure 1); Lining amino acids are all the amino acids fully encapsulating the detected channel and are divided into three classes: internal, middle, and external according to their respective positions in the layers perpendicular the channel centerline. Internal or external lining amino acids are those lying within a 5 Å distance of the start or end point, respectively, with middle amino acids constituting the remainder. A bottleneck is where the channel radius is a minimum.

## Additional files

Additional file 1:
**Description of MOLE methodology, and explanation of statistical methods used for channel analysis, Supplementary Figures S1–S4 and Supplementary Tables S1–S3.**
Additional file 2:
**Full set of channels in csv format. The MOLE 2.0 application and enzymatic data set are available for download at** http://mole.chemi.muni.cz/enzymes **.**
