# Supplementary material for: Anatomy of enzyme channels
Source: BMC Bioinformatics. 2014 Nov 18;15(1):379. doi: 10.1186/s12859-014-0379-x (PMC4245731; doi:10.1186/s12859-014-0379-x)
Supplement: Additional file 1: — Description of MOLE methodology, and explanation of statistical methods used for channel analysis, Supplementary Figures S1–S4 and Supplementary Tables S1–S3. [file 12859_2014_379_MOESM1_ESM.pdf]

Supplementary Information
to
Anatomy of Enzyme Channels

Lukáš Pravda[1,†], Karel Berka[2,†], Radka Svobodová Vařeková[1], David Sehnal[1,3], Pavel Banáš[2], Roman Laskowski[4], Jaroslav Koča[1,*], Michal Otyepka[2,*]
1 National Centre for Biomolecular Research, Faculty of Science and CEITEC - Central European Institute of Technology, Masaryk University Brno, Kamenice 5, 625 00 Brno-Bohunice, Czech Republic,
2 Regional Centre of Advanced Technologies and Materials, Department of Physical Chemistry, Faculty of Science, Palacký University Olomouc, tř. 17. listopadu 12, 771 46, Olomouc, Czech Republic.
3 Faculty of Informatics, Masaryk University Brno, Botanická 68a, 602 00 Brno, Czech Republic,
4 European Molecular Biology Laboratory European Bioinformatics Institute (EMBL-EBI), Wellcome Trust Genome Campus, Hinxton, Cambridge CB10 1SD, United Kingdom

Methods
MOLE 2.0 algorithm
The algorithm for finding channels as implemented in MOLE 2.0 (Sehnal *et al.*, 2013) involves seven steps: i) computation of the Delaunay triangulation/Voronoi diagram of the atomic centers, ii) construction of the molecular surface, iii) identification of cavities, iv) identification of possible channel start points, v) identification of possible channel end points, vi) localization of channels, and vii) filtering of the localized channels.

The Voronoi diagram divides a metric space according to the distances between discrete sets of specified objects. In our case, the objects are atomic centers with van der Waals (vdW) radii assigned according to the parm99 force field (Cornell *et al.*, 1995). Hydrogen atoms and ligands not covalently bound to the structure were deleted prior to calculation in order to enable sampling of otherwise hidden channels that could be revealed upon molecular dynamics simulations in systems with explicit hydrogens (Chovancova *et al.*, 2012) and to avoid the errors in calculation of simple Voronoi mesh in comparison to the weighted Voronoi mesh (Yaffe *et al.*, 2008) by using main protein atoms only with relatively similar size (CON).

Statistics on the frequency of aminoacids
Average frequency $p^{tunnels}$ of individual amino acids in tunnels was calculated in individual tunnels longer than 15 Å, as follows:

$$p_i^{tunnels} = \frac{\sum_{tunnels} \frac{N_i^{tunnel}}{\sum_i^{20} N_i^{tunnel}}}{N^{tunnels}} \qquad (1),$$

where N_i^{tunnel} is a number of amino acid *i* in a tunnel and $N^{tunnels}$ is the total number of tunnels. The standard deviation $\sigma^{tunnels}$ and standard errors $SE^{tunnels}$ of the mean were calculated as:

$$\sigma_i^{tunnels} = \sqrt{\frac{1}{N^{tunnels}-1}\left(\frac{\sum_{tunnels}\left(\frac{N_i^{tunnel}}{\sum_i^{20} N_i^{tunnel}}\right)^2}{N^{tunnels}} - N^{tunnels} \cdot \left(\frac{\sum_{tunnels}\frac{N_i^{tunnel}}{\sum_i^{20} N_i^{tunnel}}}{N^{tunnels}}\right)^2\right)} \qquad (2)$$

$$SE_{p_i^{tunnels}} = \frac{\sigma_i^{tunnel} \cdot F^{-1}(0.99|N^{tunnels}-1)}{\sqrt{N^{tunnels}}} \;. \qquad (3)$$

Similarly, the frequency of average amino acid composition of enzymes was calculated as:

$$p_i^{enzymes} = \frac{\sum_{enzymes} \frac{N_i^{enzyme}}{\sum_i^{20} N_i^{enzyme}}}{N^{enzymes}} \quad (4).$$

Enhancement of the frequency of amino acid in channels over average frequency of amino acid in the composition in whole enzymes (i.e. channel propensity of amino acid) was calculated as:

$$p_i^{tunnels/enzymes} = \frac{\frac{\sum_{tunnels} \frac{N_i^{tunnel}}{\sum_i^{20} N_i^{tunnel}}}{N^{tunnels}}}{\frac{\sum_{enzymes} \frac{N_i^{enzyme}}{\sum_i^{20} N_i^{enzyme}}}{N^{enzymes}}} \quad (4).$$

Note: Each lining amino acid is counted just once per channel independently on its size.

Table S1: Structural Quality of the Dataset Calculated with Molprobity (Chen *et al.*, 2010).

Clashscore	all	no channels	has channels
EC1	8.66 ± 6.85	9.44 ± 7.90	8.43 ± 6.50
EC2	9.24 ± 7.90	8.87 ± 7.52	9.42 ± 8.08
EC3	8.79 ± 7.50	7.89 ± 6.22	9.63 ± 8.44
EC4	8.62 ± 6.66	8.58 ± 6.10	8.64 ± 6.92
EC5	9.03 ± 7.03	9.37 ± 7.60	8.85 ± 6.72
EC6	11.96 ± 9.30	13.18 ± 10.78	11.20 ± 8.21
All	**8.99 ± 7.46**	**8.66 ± 7.12**	**9.17 ± 7.64**

Ramachandran outliers	all	no channels	has channels
EC1	0.44 ± 0.74	0.56 ± 1.03	0.40 ± 0.64
EC2	0.47 ± 0.83	0.43 ± 0.72	0.49 ± 0.87
EC3	0.43 ± 0.79	0.39 ± 0.69	0.47 ± 0.87
EC4	0.38 ± 0.56	0.36 ± 0.59	0.38 ± 0.54
EC5	0.38 ± 0.70	0.39 ± 0.89	0.38 ± 0.57
EC6	0.70 ± 1.06	0.92 ± 1.43	0.55 ± 0.70
All	**0.44 ± 0.78**	**0.44 ± 0.80**	**0.45 ± 0.77**

Sidechain Outlier	all	no channels	has channels
EC1	3.72 ± 3.47	4.32 ± 4.18	3.55 ± 3.23
EC2	3.69 ± 3.49	3.37 ± 3.19	3.83 ± 3.62
EC3	3.66 ± 3.48	3.47 ± 3.16	3.83 ± 3.75
EC4	3.31 ± 3.19	2.78 ± 2.95	3.55 ± 3.27
EC5	3.55 ± 3.04	3.23 ± 2.87	3.72 ± 3.12
EC6	4.96 ± 3.84	4.77 ± 4.32	5.08 ± 3.52
All	**3.68 ± 3.45**	**3.53 ± 3.36**	**3.77 ± 3.50**

MPscore	all	no channels	has channels
EC1	1.89 ± 0.49	1.95 ± 0.55	1.87 ± 0.47
EC2	1.91 ± 0.49	1.87 ± 0.48	1.93 ± 0.49
EC3	1.88 ± 0.50	1.83 ± 0.47	1.92 ± 0.53
EC4	1.87 ± 0.44	1.82 ± 0.43	1.89 ± 0.44
EC5	1.89 ± 0.47	1.87 ± 0.49	1.90 ± 0.47
EC6	2.12 ± 0.53	2.15 ± 0.58	2.11 ± 0.49
All	**1.90 ± 0.49**	**1.87 ± 0.49**	**1.92 ± 0.49**

Resolution	all	no channels	has channels
EC1	1.90 ± 0.36	1.88 ± 0.36	1.91 ± 0.36
EC2	1.96 ± 0.32	1.96 ± 0.31	1.96 ± 0.32
EC3	1.84 ± 0.38	1.83 ± 0.38	1.85 ± 0.38
EC4	1.92 ± 0.32	1.94 ± 0.30	1.91 ± 0.33
EC5	1.91 ± 0.37	1.87 ± 0.38	1.93 ± 0.36
EC6	2.12 ± 0.28	2.09 ± 0.31	2.13 ± 0.26
All	**1.91 ± 0.35**	**1.89 ± 0.36**	**1.91 ± 0.35**

Clashscore is the number of serious clashes ≥0.4 Å per 1000 atoms), Ramachandran outliers is the percentage of the backbone Ramachandran conformations outside the favored region, sidechain outliers is the percentage of sidechain conformations classed as rotamer outliers and MPscore is Molprobity score for composite metric for model quality. All of these values show that the dataset mainly contains good x-ray structures as MPscores are similar to the resolution of the X-Ray structures. (Chen *et al.*, 2010) Values also do not significantly differ between the structures without or with channels.

Results

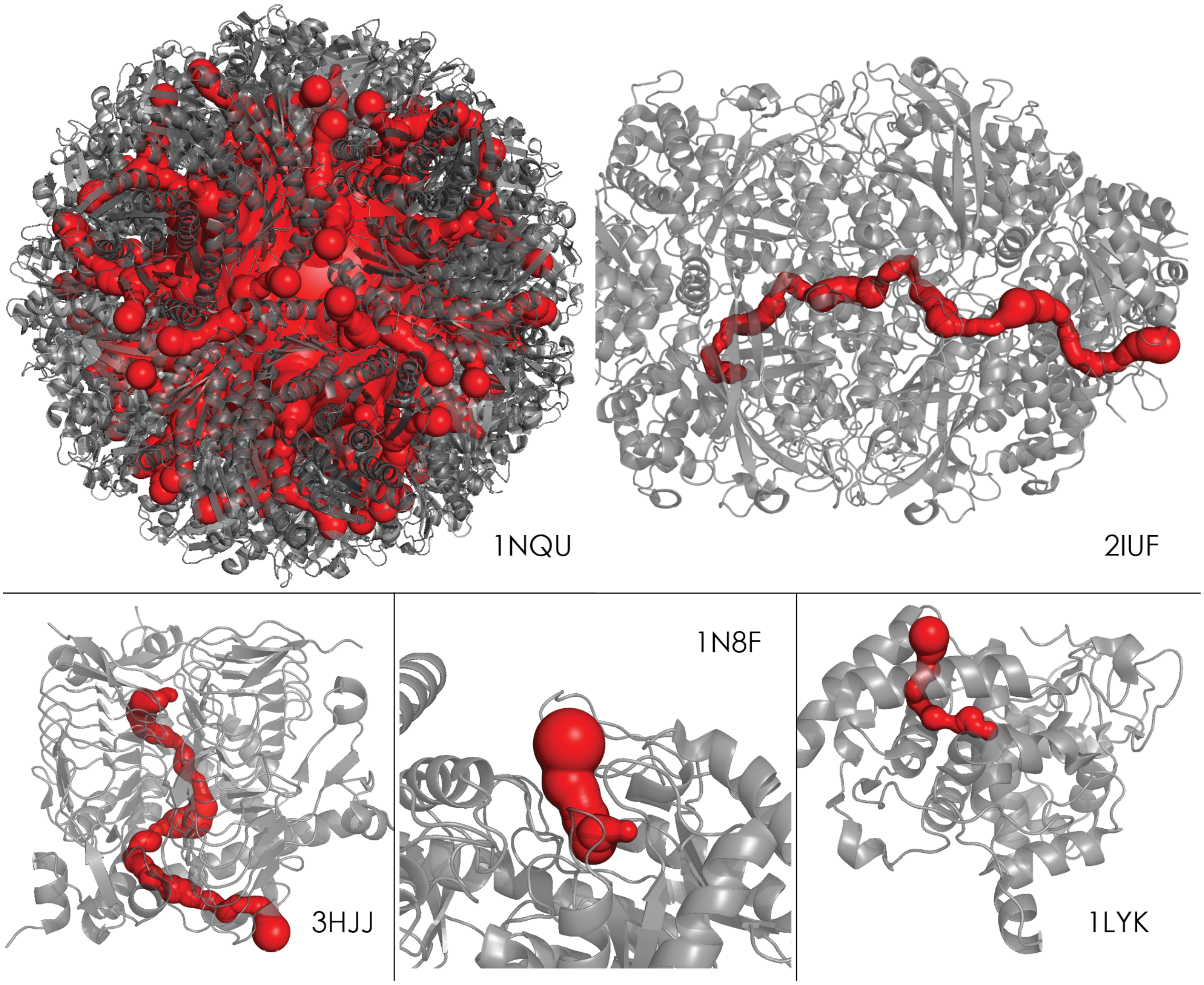

Figure S1 Proteins with Channels with Extreme Properties.
Upper left panel shows enzyme with the ***maximal number of channels*** (68) 6,7-dimethyl-8-ribityllumazine synthase from *Aquifex aeolicus* (1NQU). Upper right panel shows protein with ***the longest identified channel*** (172 Å) - Penicillium vitale catalase from *Penicillium janthinellum* (2IUF). Lower left panel shows ***small protein with the longest identified channel*** (101 Å) - maltose *O*-acetyltransferase (containing 190 lining amino acids) from *Bacillus anthracis* (3HJJ). Lower middle panel shows protein with ***the most hydrophylic channel*** - 3-Deoxy-D-arabino-heptulosonate-7-phosphate synthase (1N8F) from *Escherichia coli*. Lower right panel shows protein with ***hydrophobic channel*** – peroxidase (1LYK) from *Coprinus cinereus*.

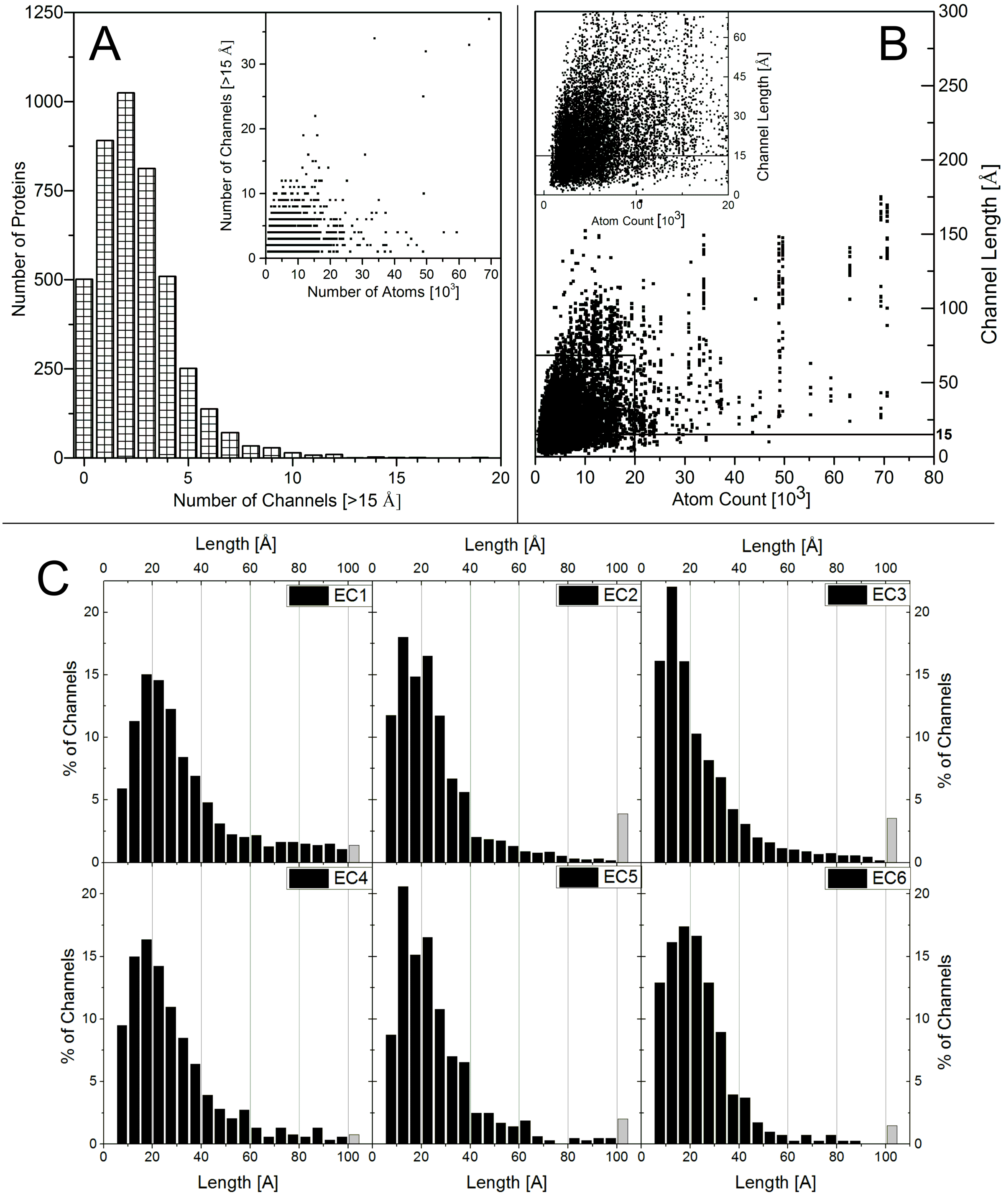

Figure S2 Channel Lengths and Counts.
Panel A shows the histogram of the number of channels in individual enzymes within CSA subset used thorough the study. The inset shows the distribution of the number of channels across the various sizes of proteins showing low significance between the number of channels and its size especially for the typical enzymatic sizes. Panel B shows the dependence of the channel lengths on the number of proteins whereas the inset zooms to the area of the more numerous smaller proteins. Horizontal line depicts the 15 Å length limit. Finally, panel C shows histograms of channel lengths for each enzymatic class. Grey column depicts percentage of channels longer than 100 Å.

Table S2. Average number of charged amino acids (N(+): positive Arg and Lys, and N(–): negative Asp and Glu) side chains lining channels, the average charge difference (Δ = N(+) – N(–)), mean and maximal range, maximal N(+) and maximal N(-), minimal and maximal Δ.

EC	N(+)	N(–)	Δ	Range		Max(+)	Max(–)	Δ	
				Mean	Max			Min	Max
EC1	2.3	1.9	0.4	4.2	27	16	19	-13	11
EC2	2.5	2.4	0.2	4.9	35	24	22	-13	19
EC3	2.0	2.4	-0.5	4.4	55	28	29	-12	10
EC4	2.5	2.3	0.2	4.7	30	20	13	-9	10
EC5	2.4	2.3	0.0	4.7	25	14	19	-16	6
EC6	2.7	2.3	0.4	5.0	31	15	18	-5	6
All	2.3	2.3	0.3	4.6	55	28	29	-16	19

Table S3. Enzymes with Channels of Extreme Hydropathy

Hydropathy	PDB	Length	EC	Hydropathy	PDB	Length	EC
3.59	1LYK	23.8	**1**.11.1.7	-3.78	1N8F	18.7	**2**.5.1.54
3.55	1BPX	22.7	**2**.7.7.7	-3.67	1SES	24.8	**6**.1.1.11
3.53	3ISC	22.7	**2**.7.7.7	-3.62	2JBM	23.2	**2**.4.2.19
3.40	3FSM	15.1	**3**.4.23.16	-3.61	3JUR	26.5	**3**.2.1.15
3.23	2IHM	21.8	**2**.7.7.7	-3.6	3K2Q	15.3	**2**.7.1.90
3.22	3LAD	16.9	**1**.8.1.4	-3.58	2CU2	15.5	**2**.7.7.13
3.04	1JHF	19.6	**3**.4.21.88	-3.58	2YXB	18.1	**5**.4.99.2
2.93	1DKU	38.9	**2**.7.6.1	-3.55	3L0G	19.5	**2**.4.2.19
2.93	1YS1	20.0	**3**.1.1.3	-3.55	1HV6	17.0	**4**.2.2.3
2.92	2O4U	19.6	**1**.3.120	-3.53	3C2E	23.48	**2**.4.2.19

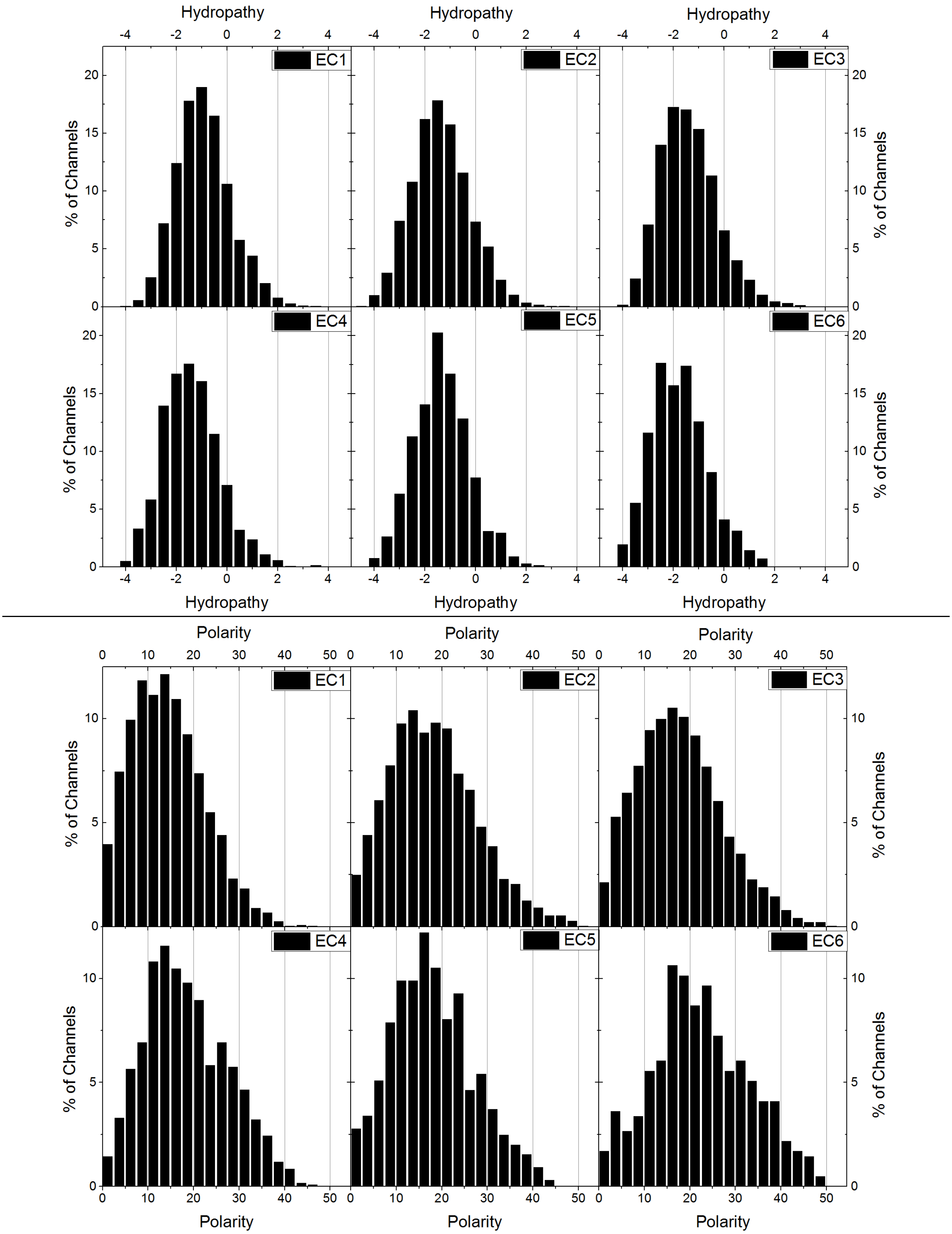

Figure S3 Histograms of Channel Hydropathy (upper) and Polarity (lower panel)

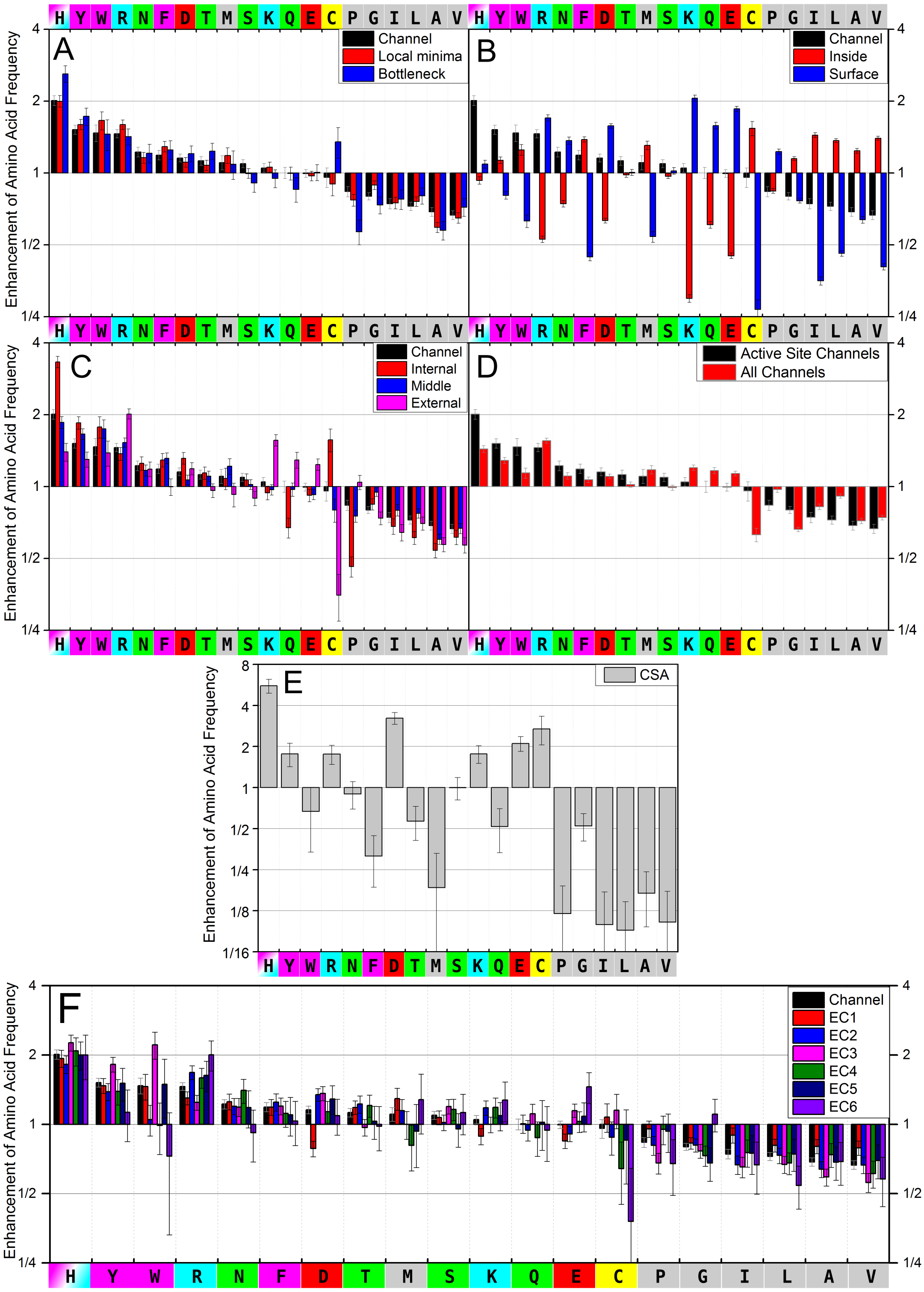

Figure S4 Comparison of amino acid propensities for individual parts of the protein structure.
Panel A shows enhancements of amino acid frequencies within channels (channel propensity) and their local minima and bottlenecks in comparison to the protein composition. The enhancement of amino acids in channels set up the order of amino acids in all graphs presented here. Panel B shows a comparison of channel propensity to the propensities for the protein surface and interior. Panel C shows comparison channel propensity to those for internal, middle and external parts of the channel. Panel D shows comparison between propensities for the channels leading to the active site and between all detectable channels found within the structures. Panel E shows catalytic site propensity of individual amino acids according to the Catalytic Site Atlas. Finally, panel F shows differences between channel propensities among individual enzymatic classes.

References

Cornell,W.D. *et al.* (1995) A Second Generation Force Field for the Simulation of Proteins, Nucleic Acids, and Organic Molecules. *J. Am. Chem. Soc.*, **117**, 5179–5197.

Chen,V.B. *et al.* (2010) MolProbity: all-atom structure validation for macromolecular crystallography. *Acta Crystallogr. D. Biol. Crystallogr.*, **66**, 12–21.

Chovancova,E. *et al.* (2012) CAVER 3.0: a tool for the analysis of transport pathways in dynamic protein structures. *PLoS Comput. Biol.*, **8**, e1002708.

Sehnal,D., *et al.* (2013) MOLE 2.0: advanced approach for analysis of biomacromolecular channels. *J. Cheminform.*, **5**, 39.

Yaffe,E. *et al.* (2008) MolAxis: Efficient and accurate identification of channels in macromolecules. *Proteins Struct. Funct. Genet.*, **73**, 72–86.
